# Ephrin Receptors (Ephs) Expression in Thymic Epithelial Tumors: Prognostic Implications and Future Therapeutic Approaches

**DOI:** 10.3390/diagnostics11122265

**Published:** 2021-12-03

**Authors:** Christos Masaoutis, Natalia Georgantzoglou, Panagiotis Sarantis, Irene Theochari, Nikolaos Tsoukalas, Mattheos Bobos, Paraskevi Alexandrou, Alexandros Pergaris, Dimitra Rontogianni, Stamatios Theocharis

**Affiliations:** 1 First Department of Pathology, National and Kapodistrian University of Athens, 11527 Athens, Greece; cmasaout@med.uoa.gr (C.M.); natalia.georgantzoglou@gmail.com (N.G.); psarantis@med.uoa.gr (P.S.); theoirene@hotmail.com (I.T.); tsoukn@yahoo.gr (N.T.); parialexandro@yahoo.gr (P.A.); alexperg@yahoo.com (A.P.); 2 Department of Pathology, Evangelismos General Hospital of Athens, 10676 Athens, Greece; dgian@otenet.gr; 3 Departament of Biomedical Science, School of Health Science, International Helenic University, Alexandrian Campus, Sindos, 57400 Thessaloniki, Greece; mbobos@icloud.com

**Keywords:** ephrin, ephrin receptor, thymoma, thymic epithelial tumor, immunohistochemistry

## Abstract

Ephrin receptors (Ephs) are receptor tyrosine kinases (RTKs) implicated in tissue development and homeostasis, and they are aberrantly expressed in tumors. Here, immunohistochemical Eph type-A and -B expression in thymic epithelial tumors (TETs) was assessed and correlated with clinicopathological parameters. Tissue microarrays from 98 TETs were stained for EphA1, -A2, -A4 -A6, -B1, -B2, -B4 and -B6. The relationship between neoplastic and lymphoid cell immunoreactivity score (H-score), histopathological parameters (Pearson’s test) and survival of 35 patients (Mantel-Cox model) was explored. Epithelial-rich subtypes showed higher EphA6 cytoplasmic H-score (B2/B3, carcinoma) (*p* < 0.001) and stronger EphA4 H-score (B3, carcinoma) (*p* = 0.011). The immature T-cells, especially in subtypes AB/B1, had higher EphB6 H-score than carcinoma-associated mature lymphocytes (*p* < 0.001); carcinomas had higher lymphocytic EphB1 H-score (*p* = 0.026). Higher lymphocytic and lower epithelial EphB6 H-score correlated with Masaoka stage ≤II (*p* = 0.043, *p* = 0.010, respectively). All cases showed variable epithelial and lymphocytic EphA2 expression, but clinicopathological associations were not reached. Our study confirmed that Eph type-A and -B expression in TETs is associated with established prognostic parameters, i.e., tumor subtype and Masaoka stage, although correlation with patient survival was not reached. Such findings suggest involvement of these RTKs in thymic neoplasia, as well as their potential utility as treatment targets.

## 1. Introduction

Thymic epithelial tumors, thymomas and thymic carcinomas, comprise a heterogenous group of neoplasms of the anterior mediastinum with a broad range of histopathological features, clinical manifestations and variable prognosis [1]. The natural course of the disease ranges from indolent to highly aggressive with extra-thoracic spread, while almost 40% of patients have para neoplastic syndromes, including myasthenia gravis and pure red cell aplasia [2].

According to the WHO classification, thymic neoplasms are categorized into types A, AB, B1-3 and C based on morphology of epithelial cells, epithelial/lymphocyte ratio and resemblance to normal thymic architecture. Prognosis worsens ranging from type A over type B to type C [1,3]. In fact, histopathological classification correlates with disease stage at diagnosis. The Masaoka–Koga staging system, the most widely accepted one, evaluates capsular invasion, invasion of adjacent tissues/organs and hematogenous and lymphogenous metastases. Approximately 80–90% of type A and B1 thymomas present as stage I–II tumors, while 50–60% of type B2 and 60–80% of type B3 and thymic carcinomas present at stage III–IV at diagnosis [3].

Ephs (after erythropoietin-producing human hepatocellular receptors) and their ligands, ephrins, comprise a complex contact-dependent communication system between cells, with a central role in normal development and physiology of a wide variety of tissues, as well as in disease pathogenesis. Ephs are a subfamily of receptor tyrosine kinases [4]. There are nine type A Ephs, EphA1–8 and EphA10, which interact with five type A ephrins, ephrin-A1–5, and five type B Ephs, EphB1–4 and EphB6, which interact with three type B ephrins, ephrin-B1–3. Eph–ephrin complexes emanate bidirectional signals that ultimately affect the cytoskeleton, cell movement and shape, cell-to-cell adhesions, proliferation and survival. Notably, depending on the cellular context and specific microenvironment, Eph–ephrin complexes can activate signaling cascades with opposite effects [5,6]. This complex and finely coordinated communication system has wide-reaching roles in the human embryogenesis and morphogenesis of several tissues: Segmentation of paraxial mesoderm (somitogenesis), neurogenesis, neuronal plasticity modulation, embryonic vascular development and spatial organization of the retina have all been linked to normal Eph–ephrin expression [6,7,8]. Additionally, emerging data have demonstrated that the Eph–ephrin bidirectional signaling is also involved in normal bone remodeling, glucose homeostasis and immune function [4,9].

Over the preceding years, an increasing number of studies has associated cancer development and progression with dysregulated Eph–ephrin expression [10]. Several Eph classes are expressed in cancer cells controlling a broad range of tumor processes by functioning as master regulators of the tumor microenvironment and either triggering or suppressing oncogenic signaling cascades. Compelling evidence suggests that Ephs are implicated in pre-metastatic niche formation by promoting angiogenesis and interfering with epithelial cell junction formation, thus enhancing invasiveness [5]. It is not surprising, therefore, that according to accumulating data, aberrant Eph expression is associated with several clinicopathological parameters crucial for patient prognosis in a variety of cancer types. Additionally, the implication of Ephs in various aspects of tumor pathogenesis has prompted the investigation of Ephs as potential therapeutic targets.

The expression of Ephs in thymic epithelial neoplasms and their potential role in their pathogenesis to our knowledge has not been described. In view of above considerations, the present study aimed to assess immunohistochemical EphA1, EphA2, EphA4, EphA6, EphB1, EphB2, EphB4 and EphB6 expression in 98 thymic epithelial tumors in association with clinicopathological parameters and patients’ survival.

## 2. Materials and Methods

### 2.1. Patient Material Collection and Patient Characteristics

**Figure 1:**
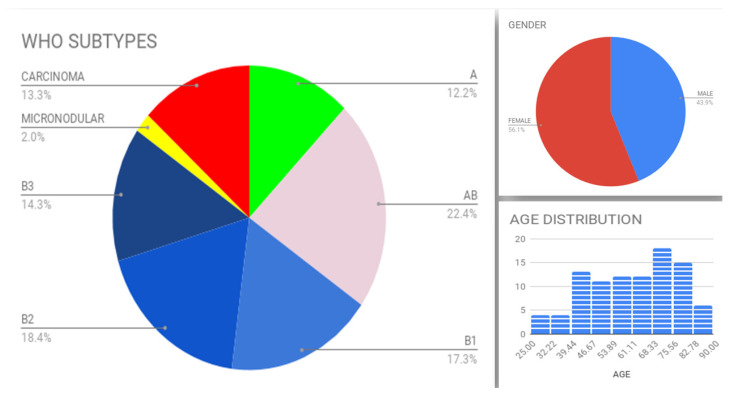
Distribution of WHO subtypes, gender and age.

Formalin-fixed paraffin-embedded (FFPE) tissue from 98 TETs resected between 2009 and 2019 was retrieved from the pathology laboratory archives of two major Athens hospitals (Evangelismos General Hospital and Laikon General Hospital, Athens, Greece). Patient characteristics are shown in Figure 1. Females were 56.1% (55/98) and males 43.9% (43/98) of the patients. The mean patient age was 61 years (range: 27–88 years). The frequency of WHO subtypes in our cohort was as follows: type A 12.2% (12/98); type AB 22.4% (22/98); type B1 17.3% (17/98); type B2 18.4% (18/98); type B3 14.3% (14/98); micronodular thymoma with lymphoid stroma (MNT) 2% (2/98); type C 13.3% (13/98). Masaoka–Koga stage was I in 19.1% (17/89); IIa in 38.2% (34/89); IIb in 16.9% (15/89); III in 19.1% (17/89); IVa in <0.1% (3/89); IVb in <0.1% (3/89) of patients. Surgical margins were negative in 70.2% (33/47) and positive in 29.8% (14/47) of cases. Co-existing myasthenia gravis was diagnosed in 58.6% (34/58) of patients, 2 of whom also suffered from pemphigus vulgaris and autoimmune thyroidopathy; among the patients without myasthenia gravis, 3 had pure red cell aplasia, and 2 had hypothyroidism. Chemotherapy was given to 26.3% (10/38) and radiotherapy to 48.6% (18/37) of patients; 5 of these patients received both chemo- and radiotherapy. Follow-up information was available for 35 patients, ranging from 5 to 134 months (average: 42.6 months); of these patients 71.4% (25/35) were alive without disease, <0.1% (3/35) were alive with disease and 0.2% (7/35) died.

### 2.2. TMA Construction

One representative FFPE tissue block from each tumor was selected after all hematoxylin–eosin (H&E)-stained slides were reviewed. TMAs were constructed using a manual tissue arrayer (TMA Model I, Beecher Instruments, Sun Prairie, WI, USA). More specifically, three to five 1.5 mm cores were transferred from each representative block into positionally encoded arrays in eight recipient paraffin blocks. Multiple cores from each single case were included to capture histological tumor heterogeneity.

### 2.3. Immunohistochemistry Procedure and Evaluation

Immunohistochemistry was carried out using standard procedures in the eight TMAs. The sections were stained with antibodies against EphA1 (clone ab37857, AbCam/at dilution 1:200); EphA2 (clone ab123877, AbCam/at dilution 1:100); EphA4 (clone D-4, Santa Cruz, CA, USA/at dilution 1:200); EphA6 (clone ab11329, AbCam/at dilution 1:250); EphB1 (5F10A4, Santa Cruz, CA, USA/at dilution 1:100); EphB2 (clone 48CT12.6.4, Santa Cruz, CA, USA/at dilution 1:74); EphB4 (clone 5G2F8, Santa Cruz, CA, USA/at dilution 1:100); EphB6 (clone 2A6B9, Santa Cruz, CA, USA/at dilution 1:100). Antigen retrieval was performed at pH 6. The Envision (Dako) visualization system was used. DAB (3,3-diaminobenzidine) was used as a chromogen and hematoxylin as counterstain. Appropriate positive controls with known Eph expression were used as previously described [11,12,13,14]. As negative control, the omitted primary antibody and substitution with an irrelevant antiserum was used.

Since no standardized immunohistochemical evaluation method for Eph expression exists in the literature, we calculated the H-score, which serves as a semiquantitative measure of immunohistochemical protein expression levels. To calculate H-score the semiquantitative staining intensity score (score 1 to 3) is multiplied by the percentage of positive cells [15]. Therefore, H-score values range between 0 and 300. The epithelial and the lymphocytic components, as well as the nuclear and cytoplasmic positivity, were separately evaluated.

### 2.4. Statistical Correlations

For the purpose of clinicopathological correlations age, gender and histological subtype according to the 2015 WHO classification were recorded for all 98 tumors. Further histological parameters (Masaoka–Koga stage and surgical margin involvement) as well as clinical parameters (co-occurrence of myasthenia gravis, co-occurrence of other autoimmune diseases, chemotherapy, radiotherapy, follow-up duration and status at last consultation) were recorded wherever available. Using the mean H-score as the cutoff for each Eph receptor, the cases were characterized as H-scorehigh and H-scorelow. The Mantel–Cox model and the Pearson’s chi-square test were applied for survival analysis and correlations with the rest of the clinicopathological parameters, respectively. SPSS Statistics software for Windows version 25.0 (Released 2017; Armonk, NY, USA: IBM Corp) was used for the statistical analysis.

### 2.5. Evaluation of Eph Expression in Normal Thymus

Eph expression in normal thymus was evaluated by retrieving data from the Human Protein Atlas Database available on www.proteinatlas.org, accessed on 16 September 2021 [16]. Since no immunohistochemical data on Ephs were available, the consensus normalized expression (NX) levels were used.

## 3. Results

### 3.1. Eph Receptors Type A

#### 3.1.1. Subcellular Topography

**Figure 2:**
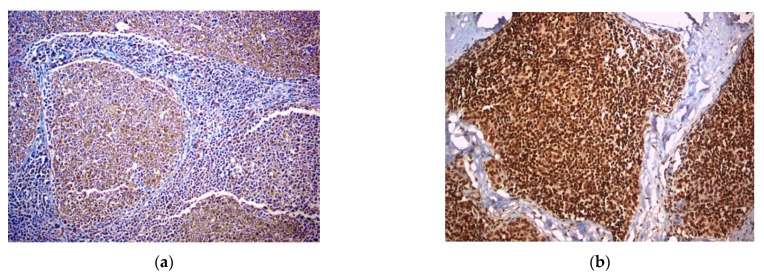

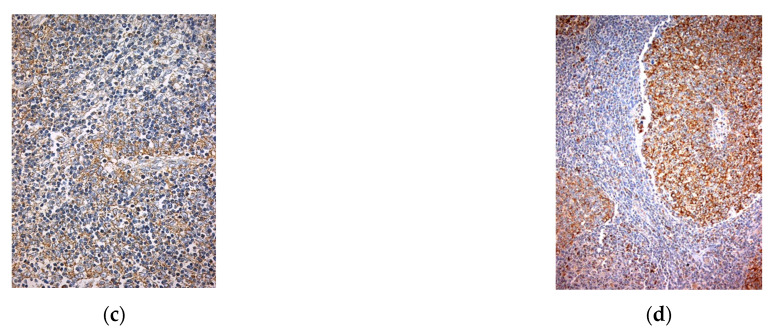
Immunohistochemical expression of EphA2 (**a**), EphA4 (**b**) and EphA6 (**c**,**d**).

EphA2 expression was exclusively cytoplasmic in both epithelial and lymphoid cells (Figure 2a). EphA4 was cytoplasmic and nuclear in the epithelial cells and nuclear in the lymphoid cells (Figure 2b). EphA6 was mainly cytoplasmic and only rarely nuclear in the epithelial cells, and it was cytoplasmic in the lymphoid component (Figure 2c,d). EphA1 was not expressed in either component (Table 1).

#### 3.1.2. Expression Levels

As evaluated by immunohistochemistry with the H-score, EphA2 expression was highly variable in both epithelial cells (mean H-score = 125; range: 5–300) and lymphocytes (mean H-score = 86; range: 0–300). EphA6 was uniformly expressed in all epithelial and lymphoid cells with only infrequent variations in staining intensity: all cases showed moderate epithelial EphA6 expression except for five strongly positive TETs—namely, one type B2, one type B3 and three type C (mean H-score = 205; range: 200–300); lymphocytic expression was strong in the majority of cases (mean H-score = 277; range: 100–300). Cytoplasmic epithelial EphA6 was variable (mean H-score = 59; range: 0–190), whereas nuclear epithelial EphA6 was very rare, being only observed in six cases (mean H-score = 3; range: 0–140); lymphocytic EphA6 was rare and generally weak (mean H-score = 6; range: 0–180).

#### 3.1.3. Clinicopathological Correlations

**Figure 3:**
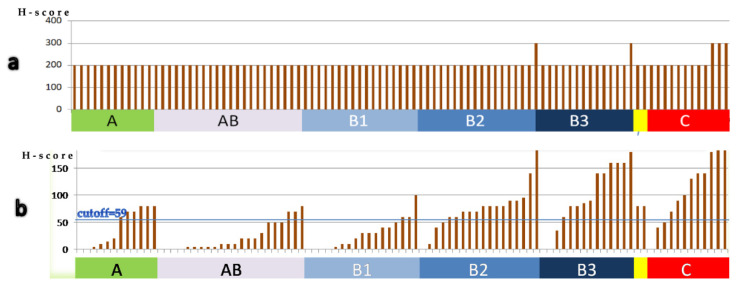
There is higher epithelial EphA4 (**a**) and cytoplasmic epithelial EphA6 (**b**) H-score in epithelial-rich subtypes. Each bar represents one case. Cases were arranged by increasing H-score in each diagram.

The more epithelial-rich TET subtypes (B2, B3 and C) presented higher cytoplasmic EphA6 H-score (*p* < 0.001) (Figure 3b); nuclear localization of EphA6 was rare and less strongly correlated with WHO classification subtypes (*p* = 0.097). Type B3 and C TETs were also more likely to intensely express EphA4 (*p* = 0.011) (Figure 3a). High lymphocytic EphA6 H-score was infrequent but more probable to be seen in the lymphocyte-rich B1 subtype (*p* = 0.015). EphA2 expression in neither the epithelial nor the lymphocytic component was statistically significantly correlated with any clinicopathological parameters; however, a tendency of type B1 TETs to have lower EphA2 expression was noted (*p* = 0.058). No correlation was found between EphA expression and Masaoka–Koga stage or survival rates.

### 3.2. Eph Receptors Type B

#### 3.2.1. Subcellular Topography

**Figure 4:**
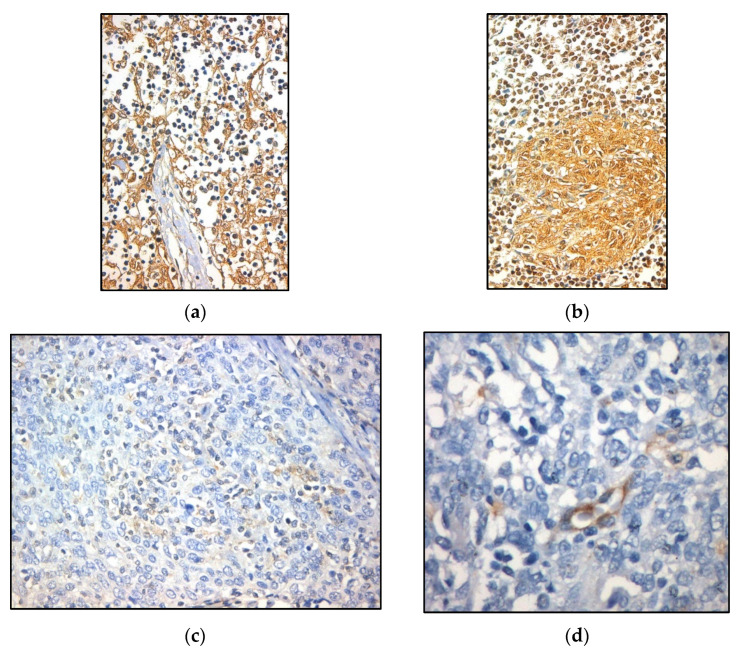

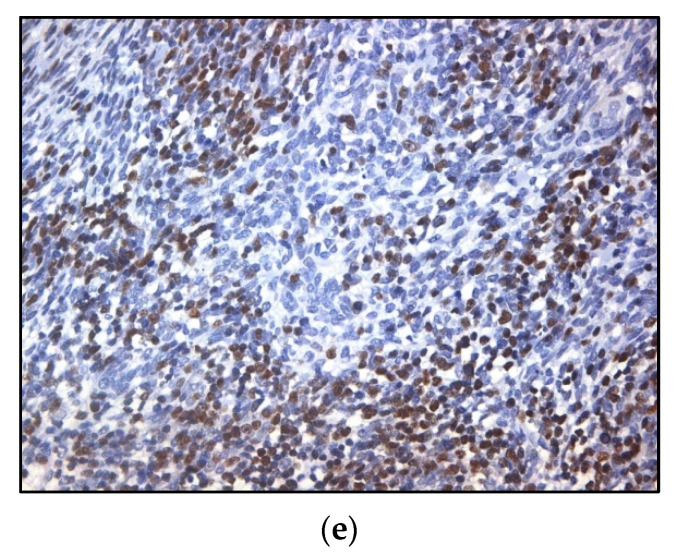
Immunohistochemical expression of EphB1 (**a**,**b**), EphB2 (**c**), EphB4 (**d**) and EphB6 (**e**).

EphB1 was positive in the cytoplasm and nuclei of epithelial cells, as well as lymphocytic nuclei (Figure 4a,b). EphB2 expression was cytoplasmic, rare and generally weak in both epithelial and lymphoid cells (Figure 4c). EphB6 was exclusively nuclear in both components, but it was more common in lymphocytes (Figure 4d). EphB4 expression was not observed in either component, but a scarce and weak endothelial expression was noted in most cases (87.7%) (Figure 4e) (Table 1).

#### 3.2.2. Expression Levels

High epithelial EphB1 expression was observed in the vast majority of cases regarding both the cytoplasmic (mean H-score = 276; range: 30–300) and the nuclear localization of the protein (mean H-score = 278; range: 60–300); lymphocytic EphB1 expression was more variable (mean H-score = 151; range: 0–300). EphB2 expression was very low in both epithelial cells (mean H-score = 2; range: 0–30) and lymphocytes (mean H-score = 6; range: 0–120, whereby the outlier H-score = 120 concerns an MNT case). EphB6 expression was low in the epithelial cells (mean H-score = 3; range: 0–29) and slightly higher in the lymphocytes (mean H-score = 18; range: 0–92).

#### 3.2.3. Clinicopathological Correlations

**Figure 5:**
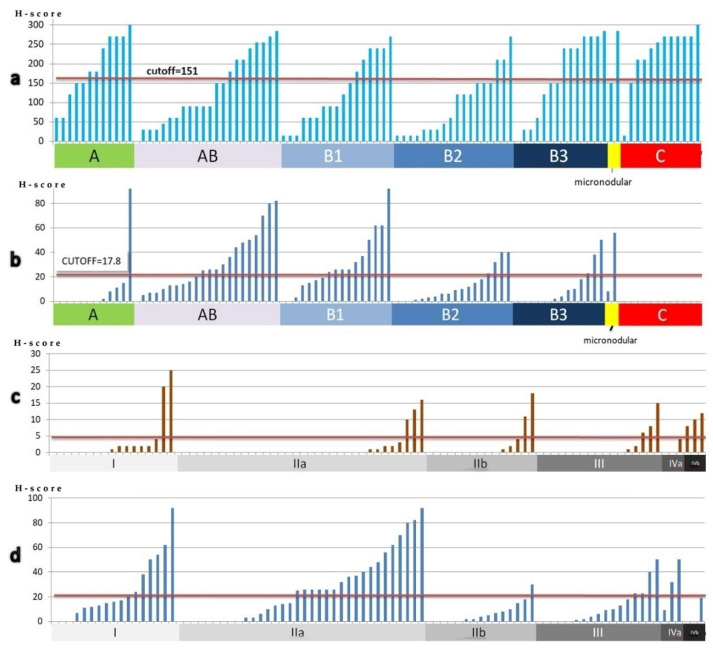
There is higher lymphocytic EphB1 H-score in thymic carcinomas (**a**) and higher lymphocytic EphB6 H-score in subtypes A and AB, but no EphB6 expression in thymic carcinomas (**b**). Lower epithelial (**c**) and higher lymphocytic (**d**) EphB6 H-scores correlate with Masaoka stage < II. Each bar represents one case. Cases were arranged by increasing H-score in each diagram.

The lymphocytic population in thymomas (especially in subtypes AB and B1), which consists of accompanying immature T cells, presented higher EphB6 H-score compared with lymphocytes in thymic carcinomas (*p* < 0.001) (Figure 5b), where they probably represent antitumoral immune reaction; conversely, lymphocytic EphB1 expression was higher in carcinomas (*p* = 0.026) (Figure 5a). Thymomas of low Masaoka stages (I and II) presented higher lymphocytic (*p* = 0.043) and lower epithelial (*p* = 0.010) EphB6 H-scores (Figure 5c,d). Lymphocytic EphB1 expression was less strongly correlated with Masaoka–Koga stage (*p* = 0.059). Correlation of EphBs expression with patients’ survival was not reached.

### 3.3. Eph Expression in Normal Thymus

**Figure 6:**
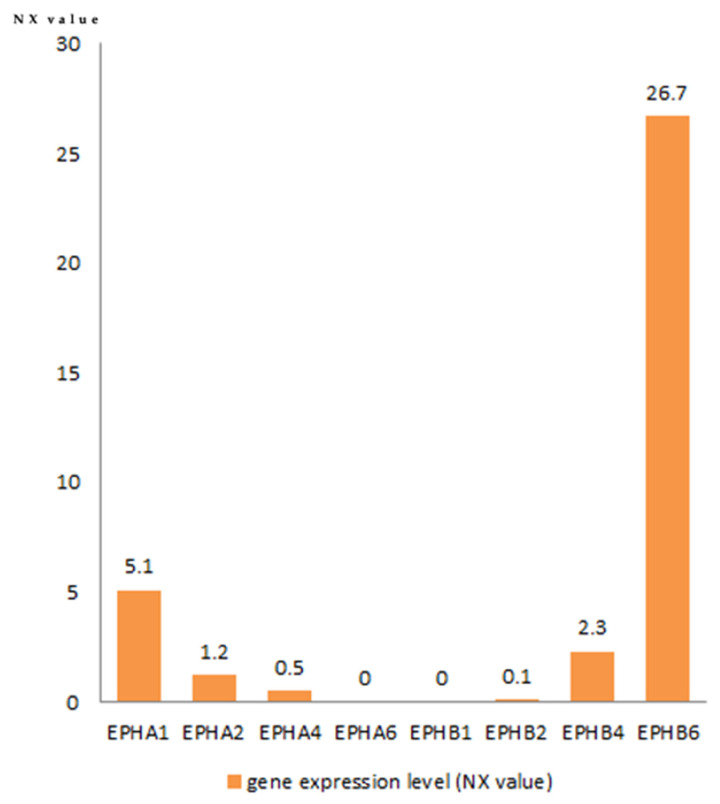
Eph expression in normal thymic tissue. Data retrieved from the Human Protein Atlas database.

The data retrieved from the Human Protein Atlas database are shown in Figure 6. EphB6 is the highest-expressed Eph in the thymus. The thymus is the fourth highest EphB6-expressing tissue in the human body following the skin, the basal ganglia and the cerebral cortex. EphA1, EphA2, EphA4, EphB2 and EphB4 expression was low. EphA6 and EphB1 are not expressed in normal thymus.

## 4. Discussion

Eph and ephrin expression has been associated with tumor pathogenesis and progression in various cancer types, rendering Ephs and ephrins promising therapeutic targets [10,17,18]. However, the role of Ephs in the tumor microenvironment of thymic neoplasms and its potential clinical applications remain largely unexplored up to now.

In this aspect, the present study provides evidence that Ephs could be implicated in the pathogenetic mechanisms underlying the neoplastic evolution in the thymus gland. Despite not reaching significant correlation with patients’ survival, Eph expression was associated with established prognostic parameters such as the tumor histologic subtype and Masaoka–Koga stage. EphA4 and EphA6 displayed higher epithelial expression in thymoma subtypes B2 and B3 as well as thymic cancer compared with subtypes A and AB (*p* = 0.011 and *p* < 0.001 for EphA4 and EphA6, respectively). EphA6 is not expressed in normal thymus according to gene expression data, but it is uniformly positive in both epithelial and lymphoid cells of all thymoma subtypes by immunohistochemistry; therefore, EphA6 could be considered a potential oncogenic driver in thymic neoplasia. EphA4 and EphA6 are implicated in tumor biology of several cancer types. Increased EphA6 expression has been described as a poor independent prognostic factor in breast cancer [19] and has been associated with disease progression in prostate cancer patients [20], where it might be implicated in angiogenesis and vascular invasion. However, the exact mechanisms with which EphA6 can potentially promote oncogenesis and disease progression remain to be fully elucidated. Similarly, EphA4 has been reported to be upregulated in pancreatic adenocarcinoma cell lines; interestingly, knocking down EphA4 expression was associated with decreased proliferating capacity [21], highlighting its potential as a therapeutic target. Miyazaki et al. described adverse clinical outcomes, such as distant metastasis and recurrence, in patients with gastric cancer and increased EphA4 expression [22]. EphA4 expression has also been associated with increased chemoresistance and radiotherapy failure in colorectal cancer patients [23,24].

In our study, the more indolent thymoma subtypes (types A and AB) were also associated with higher lymphocytic EphB6 expression in contrast with thymic cancer, which showed no expression (*p* < 0.001). Additionally, higher lymphocytic EphB6 expression correlated with lower Masaoka stage (*p* = 0.046) in contrast with epithelial EphB6, which was conversely lower in Masaoka stage < II (*p* = 0.01). These findings suggest that lymphocytic EphB6 might be implicated in the tumor microenvironment of less invasive and aggressive thymic epithelial tumors. EphB6, the highest-expressed Eph class in normal thymus, is a crucial regulator of thymocyte maturation and differentiation in the normal thymus gland, also implicated in pre-apoptotic signaling that could be important in eliminating harmful clones [25]. Loss of EphB6 expression has been associated with augmented metastatic potential in different cancer types, such as non-small cell lung cancer, melanoma and colon cancer [26,27,28]. Others, however, have linked EphB6 to increased tumor growth in breast cancer [29]. Although disruption of normal EphB6 expression in thymocytes could be implicated in the development of more aggressive phenotypes, further research is warranted in order to determine the role of lymphocytic EphB6 in the interaction of the immature lymphocytes with the neoplastic epithelial cells of thymic epithelial tumors. In the majority of cases, high epithelial and variable lymphocytic EphB1 positivity was noted, the latter being more frequent in carcinomas. Considering the absence of EphB1 expression in normal thymus, EphB1 might play a crucial role in thymic oncogenesis.

Preclinical studies on thymocyte–epithelial interactions in ephrin-deficient thymi highlight the significance of Ephs and ephrins in normal T cell maturation as well as the development and organization of the thymic epithelium. EphB-deficient mice displayed altered thymic epithelium, with more immature epithelial cell phenotypes and distorted organization characterized by the presence of epithelial cysts, areas of collapsed epithelium and distinct areas with decreased expression of epithelial markers and increased numbers of fibroblasts [30]. Alfaro et al. found decreased numbers conjugate by double-positive (CD4+ CD8+) thymocytes and thymic epithelial cells, as well as decreased TCR signaling in EphB deficient mice, highlighting the central role that Ephs and ephrins play in thymocyte–epithelial cell interactions [31,32]. It has also been demonstrated that the absence of EphB2 and EphB3 results in blockade of thymocyte maturation from the double-negative to the double-positive stage and increased numbers of apoptotic thymocytes [33]. Notably, in the present study, thymic neoplasms demonstrated very weak and rare staining for EphB2. Existing literature suggests that EphB2 is normally present in the human thymus, albeit its expression is weaker compared with mice [34]. Whether deficiency of EphB2 is implicated in thymic oncogenesis could be scope for further research. In breast cancer, EphB2 expression inhibits cell proliferation, motility and migration in vitro and has been described as a positive prognostic factor [35]. Similarly, experiments in in vivo colon cancer models demonstrated that overexpression of EphB2 is associated with decreased perfusion and suppressed growth [36].

Ephs harbor a dual role in oncogenesis, switching between tumor suppression and promotion of tumor growth and progression, depending on their ligands and specific tumor microenvironment-related factors. Ephs and ephrins emanate complex bidirectional signals through which they can suppress oncogenic pathways, such as HRAS-Erk and PI3 kinase-Akt, and inhibit proliferation, migration and metastasis, while cancer cells on the other hand employ various mechanisms to abrogate these anti-tumorigenic signals. In other instances, however, forward or reverse Eph-mediated signaling promotes cell proliferation and angiogenesis [5]. Taking into consideration the above notions, our findings suggest that Ephs and ephrins may be important regulators of the microenvironment of thymic epithelial tumor neoplasms. Their exact role (tumor suppressive or oncogenic) is highly dependent on the specific Eph class, the type of the Eph expressing cell (thymocyte or epithelial cell) and probably various other factors that remain to be determined.

Ephs have emerged as potential therapeutic targets in cancer treatment. Various strategies in order to take advantage of their tumor suppressive effect or interfere with their oncogenic properties are under evaluation in different cancer types [37]. Blocking Eph–ephrin interactions with antagonistic antibodies and peptides has been explored as a potential therapeutic strategy in melanoma, breast, colon and pancreatic cancer [38,39]. Eph-conjugated antibodies have also been examined as potential drug delivery vehicles in Eph-positive cancers [40]. Additionally, various studies provide evidence that Eph receptors could be future immunotherapy targets or could even be exploited for cancer vaccine development [41,42].

## 5. Conclusions

Expression of Eph type-A and -B in thymic epithelial neoplasms correlates with well-established prognostic factors. Understanding the complexity of the Ephs’ participation in these tumors not only can elucidate their implication in oncogenesis but also can lay the foundation for the development and application of novel therapeutic agents for this rare type of malignancy.

## Figures and Tables

**Table 1:** Subcellular topography of Ephs.

Ephs	Epithelial Cells	Lymphoid Cells
EphA1	no staining	
EphA2	cytoplasm	cytoplasm
EphA4	cytoplasm; nucleus	nucleus
EphA6	cytoplasm; nucleus	cytoplasm
EphB1	cytoplasm; nucleus	nucleus
EphB2	cytoplasm (weak)	cytoplasm (weak)
EphB4	rare weak endothelial staining	
EphB6	nucleus	nucleus

## Data Availability

The data presented in this study are available on request from the corresponding author.
